# Abstracts from the 15th International Myopia Conference: Wenzhou, P.R. China. 23-27 September 2015

**DOI:** 10.1186/s40662-016-0057-3

**Published:** 2016-11-07

**Authors:** Alexandra Benavente-Perez, Ann Nour, Tobin Ansel, Kathleen Abarr, Luying Yan, Keisha Roden, David Troilo, Chanyi Lu, Miaozhen Pan, Min Zheng, Jia Qu, Xiangtian Zhou, Christine F. Wildsoet, Fan Lu, Xiangtian Zhou, Jie Chen, Jinhua Bao, Liang Hu, Qinmei Wang, Zibing Jin, Jia Qu, Frances Rucker, Stephanie Britton, Stephan Hanowsky, Molly Spatcher, Hui-Ying Kuo, Ching-Hsiu Ke, I-Hsin Kuo, Chien-Chun Peng, Han-Yin Sun, Ian G. Morgan, Jeremy A. Guggenheim, Rupal L. Shah, Cathy Williams, Jinglei Yang, Peter S. Reinach, Sen Zhang, Miaozhen Pan, Wenfeng Sun, Bo Liu, Fen Li, Xiaoqing Li, Aihua Zhao, Tianlu Chen, Wei Jia, Jia Qu, Xiangtian Zhou, Jun Jiang, Haoran Wu, Fan Lu, Kazuo Tsubota, Hiroko Ozawa, Hidemasa Torii, Shigemasa Takamizawa, Toshihide Kurihara, Kazuno Negishi, Klaus Graef, Daniel Rathbun, Frank Schaeffel, Ladan Ghodsi, William K. Stell, Machelle T. Pardue, Ranjay Chakraborty, Han na Park, Curran S. Sidhu, P. Michael Iuvone, Michael J Collins, Nethrajeith Srinvasalu, Sally A. McFadden, Paul N. Baird, P. Michael Iuvone, Pablo Artal, Pauline Cho, SW Cheung, Pei-Chang Wu, Quan V. Hoang, Sally A. McFadden, Ranjay Chakraborty, Duk C. Lee, Erica G. Landis, Michael A. Bergen, Curran Sidhu, Samer Hattar, P. Michael Iuvone, Richard A. Stone, Machelle T. Pardue, Ravi Metlapally, Ruiqin Li, Qinglin Xu, Hong Zhong, Chenglin Pan, Weizhong Lan, Xiaoning Li, Ling Chen, Zhikuan Yang, Scott A. Read, Seang-Mei Saw, Shi-Jun Weng, Xiao-Hua Wu, Kang-Wei Qian, Yun-Yun Li, Guo-Zhong Xu, Furong Huang, Xiangtian Zhou, Jia Qu, Xiong-Li Yang, Yong-Mei Zhong, Earl L Smith, Baskar Arumugam, Li-Fang Hung, Lisa A. Ostrin, Klaus Trier, Monica Jong, Brien A. Holden, Thomas Chuen Lam, Samantha Shan, Bing Zuo, Sally A. McFadden, Dennis Yan-yin Tse, Jingfang Bian, King-Kit Li, Quan Liu, Chi-ho To, Timothy J. Gawne, John T. Siegwart, Alexander H. Ward, Thomas T. Norton, Xiangtian Zhou, Yan Zhang, Yue Liu, Carol Ho, Eileen Phan, Abraham Hang, Emily Eng, Christine Wildsoet

**Affiliations:** 1 Department of Biological Sciences, SUNY College of Optometry, New York, NY 10036 USA; 2 School of Optometry and Ophthalmology and Eye Hospital, Wenzhou Medical University, Wenzhou, Zhejiang 325027 China; 3 Center for Eye Disease & Development, School of Optometry & Vision Science Program, University of California Berkeley, Berkeley, CA 94720-2020 USA; 4 Wenzhou Medical University, Wenzhou, Zhejiang 325027 China; 5 Biomedical Science and Disease, New England College of Optom, Boston, MA USA; 6 School of Optometry, Chung Shan Medical University, Taichung City, 402 Taiwan; 7 Institute of Medicine, Chung Shan Medical University, Taichung City, 402 Taiwan; 8 Division of Biochemistry and Molecular Biology, Research School of Biology, Australian National University, Canberra, Australia; 9 State Key Laboratory of Ophthalmology and Division of Preventive Ophthalmology, Zhongshan Ophthalmic Center, Sun Yat-Sen University, Guangzhou, China; 10 School of Optometry & Vision Sciences, Cardiff University, Cardiff, UK; 11 School of Social and Community Medicine, University of Bristol, Bristol, UK; 12 School of and Eye Hospital, Wenzhou Medical University, Wenzhou, Zhejiang 325027 China; 13 State Key Laboratory Cultivation Base and Key Laboratory of Vision Science, Ministry of Health and Zhejiang Provincial Key Laboratory of Ophthalmology and Optometry, Wenzhou, Zhejiang 325027 People’s Republic of China; 60 Shanghai Key Laboratory of Diabetes Mellitus and Center for Translational Medicine, Shanghai Jiao Tong University Affiliated Sixth People’s Hospital, Shanghai, 200233 China; 14 Eye Hospital of Wenzhou Medical University, Wenzhou, Zhejiang 325027 China; 15 Department of Ophthalmology, Keio University, School of Medicine, Shinjuku-ku, Tokyo, 160-8582 Japan; 16 General Medicine, Ichikawa Medical Center, Urayasu City, Chiba 279-0001 Japan; 17 Ophthalmic Research Institute, University of Tuebingen, 72076 Tuebingen, Germany; 18 Bernstein Center for Computational Neuroscience, Tuebingen, 72076 Tuebingen Germany; 19 Werner Reichardt Centre for Integrative Neuroscience, Otfried-Mueller-Straße 25, Tuebingen, 72076 Tuebingen Germany; 20 O’Brien Centre for the Bachelor of Health Sciences, Cumming School of Medicine, University of Calgary, Calgary, Alberta Canada; 21 Departments of Cell Biology and Anatomy, and Surgery, Cumming School of Medicine, University of Calgary, Calgary, Alberta Canada; 22 Atlanta VA Center for Visual and Neurocognitive Rehabilitation, Decatur, GA 30033 USA; 23 Department of Biomedical Engineering, Georgia Institute of Technology, Atlanta, GA 30332 USA; 24 Ophthalmology, Emory University, Atlanta, GA 30322 USA; 25 Pharmacology, Emory University, Atlanta, GA 30322 USA; 26 School of Optometry and Vision Science, Queensland University of Technology, Brisbane, Queensland 4059 Australia; 27 Centre for Eye Research Australia, University of Melbourne, Melbourne, Victoria Australia; 28 School of Psychology, University of Newcastle, Callaghan, Newcastle, NSW Australia; 29 Departments of Ophthalmology and Pharmacology, Emory University School of Medicine, Atlanta, GA 30322 USA; 30 Laboratorio de Óptica, Universidad de Murcia, Campus de Espinardo, 30100 Murcia, Spain; 31 School of Optometry, The Hong Kong Polytechnic University, Hong Kong SAR, China; 32 Department of Ophthalmology, Kaohsiung Chang Gung Memorial Hospital, and Chang Gung University College of Medicine, Kaohsiung, Taiwan; 33 Department of Ophthalmology, Columbia University College of Physicians and Surgeons, New York, NY 10032 USA; 34 School of Psychology & Hunter Medical Research Institute, The University of Newcastle, Callaghan, NSW 2308 Australia; 35 Ophthalmology, Emory University School of Medicine, Atlanta, GA 30307 USA; 36 Rehab R&D Center of Excellence, Atlanta VA Medical Center, Decatur, GA 30033 USA; 37 Biology and Neuroscience, Johns Hopkins University, Baltimore, MD 21218 USA; 38 Pharmacology, Emory University, Atlanta, GA 30322 USA; 39 Ophthalmology, University of Pennsylvania School of Medicine, Philadelphia, PA 19104 USA; 40 Wallace H. Coulter Department of Biomedical Engineering, Georgia Institute of Technology, Atlanta, GA 30332 USA; 41 School of Optometry, University of California, Berkeley, CA 94720 USA; 42 Aier School of Ophthalmology, Central South University, Changsha, Hunan, China; 43 Optometry Visual Science Institute, Aier Eye Hospital Group, Changsha, Hunan, China; 44 Changsha Aier Eye Hospital, Changsha, Hunan, China; 46 School of Optometry and Vision Science, Queensland University of Technology, Brisbane, Queensland 4059 Australia; 47 Myopia Research Unit, Singapore Eye Research Institute, Singapore, Singapore; 48 Saw Swee Hock School of Public Health, National University of Singapore, Singapore, Singapore; 49 Institute of Neurobiology, Institutes of Brain Science, State Key Laboratory of Medical Neurobiology and Collaborative Innovation Center for Brain Science, Fudan University, Shanghai, 200032 China; 50 School of Ophthalmology and Optometry and Eye Hospital, Wenzhou Medical University, Wenzhou, Zhejiang 325035 China; 51 University of Houston College of Optometry, Houston, TX 77204 USA; 52 Brien Holden Vision Institute, Sydney, Australia; 53 Trier Research Laboratories, Tingskiftevej 6, 2900 Hellerup, Denmark; 54 Laboratory of Experimental Optometry, Centre for Myopia Research, School of Optometry, The Hong Kong Polytechnic University, Hung Hom, Hong Kong, China; 55 State Key Laboratory of Ophthalmology, Zhongshan Ophthalmic Center, Sun Yat-Sen University, Guangzhou, China; 56 School of Psychology, The University of Newcastle, Faculty of Science and IT, Callahgan, New South Wales Australia; 57 Department of Optometry and Vision Science, University of Alabama at Birmingham (UAB), Birmingham, AL 35294 USA; 58 School of Ophthalmology and Optometry, Wenzhou Medical University, Wenzhou, Zhejiang 325027 China; 59 School of Optometry, University of California at Berkeley, Berkeley, CA 94720 USA

## Abstract

O1 Changes in peripheral refraction associated with decreased ocular axial growth rate in marmosets

Alexandra Benavente-Perez, Ann Nour, Tobin Ansel, Kathleen Abarr, Luying Yan, Keisha Roden, David Troilo

O2 PPARα activation suppresses myopia development by increasing scleral collagen synthesis--a new drug target to suppress myopia development

Chanyi Lu, Miaozhen Pan, Min Zheng, Jia Qu, Xiangtian Zhou

O3 Evidence and possibilities for local ocular growth regulating signal pathways

Christine F Wildsoet

O4 Myopia researches at Eye Hospital of Wenzhou Medical University

Fan Lu, Xiangtian Zhou, Jie Chen, Jinhua Bao, Liang Hu, Qinmei Wang, Zibing Jin, Jia Qu

O5 Color, temporal contrast and myopia

Frances Rucker, Stephanie Britton, Stephan Hanowsky, Molly Spatcher

O6 The impact of atropine usage on visual function and reading performance in myopic school children in Taiwan

Hui-Ying Kuo, Ching-Hsiu Ke, I-Hsin Kuo, Chien-Chun Peng, Han-Yin Sun

O7 Increased time outdoors prevents the onset of myopia: evidence from randomised clinical trials

Ian G Morgan

O8 Environmental risk factors and gene-environment interactions for myopia in the ALSPAC cohort

Jeremy A. Guggenheim, Rupal L. Shah, Cathy Williams

O9 Retinal metabolic profiling identifies declines in FP receptor-linked signaling as contributors to form-deprived myopic development in guinea pigs

Jinglei Yang, Peter S. Reinach, Sen Zhang, Miaozhen Pan, Wenfeng Sun, Bo Liu, Xiangtian Zhou

O10 The study of peripheral refraction in moderate and high myopes after one month of wearing orthokeratology lens

Jun Jiang, Haoran Wu, Fan Lu

O11 Axial length of school children around the earth’s equatorial area and factors affecting the axial length

Kazuo Tsubota, Hiroko Ozawa, Hidemasa Torii, Shigemasa Takamizawa, Toshihide Kurihara, Kazuno Negishi

O12 Processing of defocus in the chicken retina by retinal ganglion cells

Klaus Graef, Daniel Rathbun, Frank Schaeffel

O13 Blue SAD light protects against form deprivation myopia in chickens, by local signaling within the retina

Ladan Ghodsi, William K. Stell

O14 Contributions of ON and OFF pathways to emmetropization and form deprivation myopia in mice

Machelle T. Pardue, Ranjay Chakraborty, Han na Park, Curran S. Sidhu, P. Michael Iuvone

O15 Response of the human choroid to defocus

Michael J Collins

O16 What can RNA sequencing tell us about myopic sclera?

Nethrajeith Srinvasalu, Sally A McFadden, Paul N Baird

O17 Overview of dopamine, retinal function, and myopia

P. Michael Iuvone

O18 The eye as a "robust" optical system and myopia

Pablo Artal

O19 Effect of discontinuation of orthokeratology lens wear on axial elongation in children

Pauline Cho, SW Cheung

O20 Myopia prevention in Taiwan

Pei-Chang Wu

O21 Alternatives to ultraviolet light and riboflavin for in vivo crosslinking of scleral collagen

Quan V. Hoang, Sally A. McFadden

O22 Absence of intrinsically photosensitive retinal ganglion cells (ipRGC) alters normal refractive development in mice

Ranjay Chakraborty, Duk C. Lee, Erica G. Landis, Michael A. Bergen, Curran Sidhu, Samer Hattar, P. Michael Iuvone, Richard A. Stone, Machelle T. Pardue

O23 Scleral micro-RNAs in myopia development and their potential as therapeutic targets

Ravi Metlapally

O24 Effects of the long-wavelength filtered continuous spectrum on emmetropization in juvenile guinea pigs

Ruiqin Li, Qinglin Xu, Hong Zhon, Chenglin Pan, Weizhon Lan, Xiaoning Li, Ling Chen, Zhikuan Yang

O25 Ocular and environmental factors associated with eye growth in childhood

Scott A. Read

O26 Overview- prevention and prediction of myopia and pathologic myopia

Seang-Mei Saw

O27 New insights into the roles of retinal dopamine in form-deprivation myopia and refractive development in C57BL/6 mice

Shi-Jun Weng, Xiao-Hua Wu, Kang-Wei Qian, Yun-Yun Li, Guo-Zhong Xu, Furong Huang, Xiangtian Zhou, Jia Qu, Xiong-Li Yang, Yong-Mei Zhong

O28 The effects of the adenosine antagonist, 7-methylxanthine, on refractive development in rhesus monkeys

Earl L Smith III, Baskar Arumugam, Li-Fang Hung, Lisa A. Ostrin, Klaus Trier, Monica Jong, Brien A. Holden

O29 Application of SWATH™ based next generation proteomics (NGP) in studying eye growth: opportunities and challenges

Thomas Chuen Lam, Bing Zuo, Samantha Shan, Sally A. McFadden, Dennis Yan-yin Tse, Jingfang Bian, King-Kit Li, Quan Liu, Chi-ho To

O30 How could emmetropization make use of longitudinal chromatic aberration?

Timothy J. Gawne, John T. Siegwart Jr., Alexander H. Ward, Thomas T. Norton

O31 Balance effect of dopamine D1 and D2 receptor subtype activation on refraction development

Xiangtian Zhou

O32 BMP gene expression changes in chick rpe in response to visual manipulations

Yan Zhang, Yue Liu, Carol Ho, Eileen Phan, Abraham Hang, Emily Eng, Christine Wildsoet

## O1 Changes in peripheral refraction associated with decreased ocular axial growth rate in marmosets

### Alexandra Benavente-Perez, Ann Nour, Tobin Ansel, Kathleen Abarr, Luying Yan, Keisha Roden, David Troilo

#### Department of Biological Sciences, SUNY College of Optometry, New York, NY, 10036, USA

##### **Correspondence:** Alexandra Benavente-Perez (abenavente@sunyopt.edu)

**Purpose**

To assess the peripheral refractive changes that occur when eye growth decelerates in marmosets.

**Methods**

We measured peripheral refraction and on-axis vitreous chamber depth on a total of 53 marmosets; 36 treated monocularly for 12 weeks 9 hrs/day (26 with -5 D contact lenses, 10 with +5 D) and 17 untreated controls. From the 26 marmosets treated with -5 D, 10 wore contact lenses 9 hrs/day without interruptions and were measured at the end of 10 weeks of treatment (T10) and after 4 weeks of recovery (R14). Eight had both contact lenses removed for 30 mins twice/day (mid-morning and mid-afternoon) during the first four weeks of treatment (early interruption group) and eight had the lenses removed during the second four weeks of treatment (late interruption group). The two interruption groups were measured after 4, 8 and 12 weeks of treatment only (T4, T8, T12).

**Results**

When compared with marmosets with no interruption or late interruption, reduced ocular axial growth rate was seen in: (1) marmosets treated with +5 D; (2) marmosets interrupted during the first 4 weeks of treatment; (3) the recovery period of marmosets treated with -5 D compared (all p < 0.05). These eyes with significantly lower overall axial growth rates also exhibited less peripheral hyperopia on the nasal retina than marmosets with late interruption or continuous wear (p < 0.05), whereas marmosets treated with +5 D had similar axial and peripheral refractive profiles than untreated eyes (p > 0.05). In addition, the rate of axial eye growth in the continuous wear group regressed during the 4-week recovery period (p < 0.001) and became relatively more myopic on the nasal retina compared with their peripheral refractive state after treatment at the end of recovery (p < 0.01).

**Conclusion**

Relative peripheral refraction (RPR) changes with induced changes in axial eye growth. In previous studies we described how induced increases in axial growth rate changed the RPR towards relative hyperopia. We now report that eyes with slower axial growth rates during emmetropization, lens treatment or recovery from visual compensation, exhibit RPR changes towards relative myopia. These results, combined with previous results from our lab, suggest that peripheral refraction in the marmoset can become more hyperopic or myopic relative to axial refraction and it correlates with changes in axial eye growth. The changes are likely a consequence of induced axial changes, but may be a contributing visual factor in the further development of axial growth and myopia.

## O2 PPARα activation suppresses myopia development by increasing scleral collagen synthesis--a new drug target to suppress myopia development

### Chanyi Lu, Miaozhen Pan, Min Zheng, Jia Qu, Xiangtian Zhou

#### School of Optometry and Ophthalmology and Eye Hospital, Wenzhou Medical University, Wenzhou, Zhejiang, 325027, China

##### **Correspondence:** Xiangtian Zhou (zxt@mail.eye.ac.cn)

**Aims**

Myopia incidence is markedly increasing. Nearly 80 % of the individuals in some populations are afflicted with this sight-compromising problem. There are some indications that the peroxisome proliferator activated receptor subtype, PPARα, may contribute to this processes because one of its effects includes reducing extracellular matrix elaboration during anti-fibrosis. Furthermore, a PPARα agonist also inhibited lens-induced form deprived (FD) myopia development in chickens. Thus, in the current work, we determined if: 1) Refractive error development in homozygous PPARα knockout mice is different than that in their wild-type (WT) counterparts; 2) the loss of PPARα function can affect scleral collagen expression; 3) peribulbar PPARα agonist injections of either Clofibrate, Fenofibrate or Bezafibrate in FD myopic guinea pigs alters this condition.

**Methods**

(1) Refractive development in homozygous and heterozygous PPARα-knockout and WT littermate mice was measured at 4, 6 and 8 weeks of age. Refraction was measured with an eccentric infrared photorefractor. Corneal thickness, anterior chamber depth, lens thickness, vitreous chamber depth (VCD) and axial length (AL) were evaluated using optical coherence tomography.

(2) The expression of collagen type I was detected by Western Blot. Four pieces of sclera from two mice were pooled together as one sample. The total protein was extracted by RIPA strong lysis buffer, broken down using ball mill with three stainless steel bead per tube at 30 Hz for 10 minutes, then sonicated at 200 Hz for 5 seconds with 3 cycles at an interval of 3 seconds per cycle. 100 μg protein sample was loaded on each sample well and hybridized with antibody.

(3) Three-week old pigmented guinea pigs were randomly divided into normal vision and form deprived myopia (FDM) groups. The FDM group was subdivided into: a) non-injection group; b) vehicle group and c) drug-treated group. Peribulbar injections were given daily for 4 weeks: the vehicle group received DMSO whereas the drug-treated groups received either Clofibrate, Fenofibrate, or Bezafibrate. Refraction and ocular biometric parameters were measured in both eyes of individual animals at 0, 2 and 4 weeks after drug treatment. Refraction was measured with an eccentric infrared photoretinoscope. Ocular dimensions were measured with an A-scan ultrasonograph.

**Results**

1. PPARα-knockout mice developed relatively more myopia compared with their WT littermates and heterozygous counterparts. Furthermore, refractive error progression in heterozygous PPARα knockout mice was intermediate between that in knockout mice and WT counterparts at every time point (4 weeks: PPARα-knockout mice -17.96 ± 1.98 D vs. heterozygous counterparts -10.35 ± 1.10 D vs. WT counterparts -6.68 ± 1.37 D; 6 weeks: PPARα-knockout mice -13.01 ± 2.10 D vs. heterozygous counterparts -5.37 ± 1.42 D vs. WT counterparts -1.30 ± 1.06 D; 8 weeks: PPARα-knockout mice -9.44 ± 2.46 D vs. heterozygous counterparts -3.28 ± 1.56 D vs. WT counterparts -1.95 ± 0.87 D). The AL of the homozygous knockouts was significantly shorter than that of their wild-type counterparts. On the other hand, in the heterozygous mice the AL change was intermediate between that in homozygous mice and WT littermates (4 weeks: PPARα-knockout mice 2.503 ± 0.016 mm vs. heterozygous counterparts 2.530 ± 0.012 mm vs. WT counterparts 2.579 ± 0.012 mm; 6 weeks: PPARα-knockout mice 2.584 ± 0.018 mm vs. heterozygous counterparts 2.623 ± 0.015 mm vs. WT counterparts 2.656 ± 0.012 mm; 8 weeks: PPARα-knockout mice 2.666 ± 0.021 mm vs. heterozygous counterparts 2.675 ± 0.011 mm vs. WT counterparts 2.708 ± 0.013 mm).
2. We compared the expression of sclera collagen type I between PPARα-knockout mice and their WT littermates at 4 weeks old. Collagen expression in the sclera was decreased by 26 percent in PPARα-knockout mice compared with their WT counterparts (P < 0.05).
3. Peribulbar injection of either Clofibrate, Fenofibrateor or Bezafibrate inhibited myopia development in all cases (FDM + Clofibrate 2 ng vs. FDM + DMSO: -7.96 ± 2.41 D vs. -10.62 ± 2.55 D, P < 0.05; FDM + Fenofibrate 9 ng vs. FDM + DMSO: -6.62 ± 2.96 D vs. -9.05 ± 2.19 D, P < 0.05; FDM + Bezafibrate 3.5 ng vs. FDM + DMSO: -6.14 ± 1.86 D vs. -9.14 ± 2.37 D, P < 0.05). The progressive increases in VCD and AL were also suppressed. The three fibrates, however, did not alter the refraction and AL in any of the normal vision subgroups (P > 0.05).

**Conclusions**

Myopia induced by PPARα dysfunction indicated that reduced activation of PPARα may participate in myopia formation by contributing to the regulation of scleral collagen type I expression. Fibrates can be a potential therapeutic candidate for reducing myopia development since they selectively inhibited FDM development without altering refraction and AL in the normal vision group.

## O3 Evidence and possibilities for local ocular growth regulating signal pathways

### Christine F Wildsoet (wildsoet@berkeley.edu)

#### Center for Eye Disease & Development, School of Optometry & Vision Science Program, University of California Berkeley, Berkeley, CA 94720-2020, USA

**Purpose**

Interest in the mechanisms underlying eye growth regulation has risen steeply with the rapid rise in the prevalence of myopia, as an example of deregulated eye growth. This presentation seeks to review the evidence for local ocular growth regulation as an introduction to the presentations that follow.

**Methods**

Evidence will be summarized from studies, either directly investigating the signaling pathways regulating ocular growth, or providing indirect evidence for local ocular control. Studies involving neural lesioning, including optic nerve section in the chick and guinea pig and an early study involving chiasmal lesioning in the monkey are considered, along with studies involving local (regional) manipulations of the retinal image. Plausible mechanisms by which retinal signals may be relayed by the retinal pigment epithelium (RPE) are also briefly reviewed.

**Results**

Results from neural lesioning studies suggest that ocular growth is largely under local control, although there are subtle behavioral differences introduced by isolating eyes from the brain by optic nerve section that require explanation and there are apparent species differences in the effects of chiasmal lesioning. Nonetheless, local form deprivation and optical defocus manipulations produce ocular growth changes that are confined to the affected segment of the vitreous chamber, with local ocular regulation providing the most parsimonious explanation. The latter requires that local signals generated within the retina must be relayed to the adjacent choroid and sclera, via the RPE. Such signals may be relayed via altered movement of ions and/or secretion of growth factors by the RPE.

**Conclusion**

Evidence for local ocular growth regulation is strong and recent research has also provided some insights into how retinal signals may be relayed to the choroid and sclera. Such data add credibility to the notion that myopia can be controlled by local optical manipulations, and open the possibility of novel pharmacological interventions for controlling myopia.

## O4 Myopia researches at Eye Hospital of Wenzhou Medical University

### Fan Lu, Xiangtian Zhou, Jie Chen, Jinhua Bao, Liang Hu, Qinmei Wang, Zibing Jin, Jia Qu

#### Wenzhou Medical University, Wenzhou, Zhejiang, 325027, China

##### **Correspondence:** Fan Lu (lufan@mail.eye.ac.cn)

A series of myopia related research projects have been organized by the Eye Hospital of WMU with significant outcomes. The projects include: 1) screening programs, covering a variety of subject groups such as the newborn baby, kindergartens, primary schools. The screening regions cover the local communities and Tibet’s highland. 2) Clinical studies focusing on myopia correction studies through refractive surgery, and students’ myopia intervention studies through lenses. 3) Laboratory-based *in vitro* and *in vivo* biological studies for exploring the mechanism of myopia development and intervention.

## O5 Color, temporal contrast and myopia

### Frances Rucker, Stephanie Britton, Stephan Hanowsky, Molly Spatcher

#### Biomedical Science and Disease, New England College of Optom, Boston, MA, USA

##### **Correspondence:** Frances Rucker (ruckerf@neco.edu)

**Purpose**

It has been shown that blue light protects the eye against myopigenic changes in refraction [1]. Chicks exposed to luminance flicker in the form of a flickering yellow light (without a blue light component) showed a hyperopic shift at high temporal frequencies and a myopic shift at low temporal frequencies, while those exposed to white light (with a blue light component) maintained a constant refraction. In this experiment, we examine the temporal and blue light sensitivity of the emmetropization mechanism to color flicker.

**Methods**

White Leghorn chicks (4-5 day old) were exposed (9 am to 5 pm) to sinusoidal color modulation (0.8) at one of six temporal frequencies (0, 0.2, 1, 2, 5, 10 Hz) over 3 days and were kept in a dark chamber at night. Red/green color flicker (without blue) consisted of counter-phase modulation of red and green, while blue/yellow color flicker (with blue) consisted of in-phase modulation of red and green light and counterphase modulation of blue light (all conditions: mean 680 lux). Pre- and post- measurements of ocular components and refraction were made with a non-contact ocular biometer (Lenstar LS 900) and a Hartinger Coincidence Refractometer.

**Results**

Low temporal frequencies and red/green color flicker produced myopigenic changes in eye length, vitreous chamber depth and choroid (ANOVA: p < 0.02 all). With red/green flicker, there was more eye growth at low frequencies than with blue/yellow flicker (p < 0.001). With red/green flicker, eyes were 110 μm longer at 0.2 Hz than they were at 10 Hz (p < 0.02), while with blue/yellow flicker, they were only 48 μm longer. With red/green flicker, choroids were thinner at intermediate temporal frequencies (p = 0.03). At 5 Hz, choroids thinned 60 μm more with red/green flicker, than with blue/yellow flicker (p = 0.03). There were no significant changes in refraction with frequency and illumination condition.

**Conclusions**

With color flicker, the presence of blue light protected against temporal frequency dependent myopigenic changes in ocular components.

1. Rucker F, Britton S, Spatcher M, Hanowsky S. Blue Light Protects Against Temporal Frequency Sensitive Refractive Changes. Invest Ophthalmol Vis Sci. 2015;56(10):6121-31.

## O6 The impact of atropine usage on visual function and reading performance in myopic school children in Taiwan

### Hui-Ying Kuo^1^, Ching-Hsiu Ke^1^, I-Hsin Kuo^1^, Chien-Chun Peng^2^, Han-Yin Sun^1^

#### ^1^School of Optometry, Chung Shan Medical University, Taichung City, 402 Taiwan; ^2^Institute of Medicine, Chung Shan Medical University, Taichung City, 402 Taiwan

##### **Correspondence:** Han-Yin Sun (hanying@csmu.edu.tw)

**Purpose**

To investigate the effects of atropine usage on changes in ocular physiology, binocular visual function and near work performance in myopic Taiwanese children.

**Methods**

1305 school children aged 7 to 12 years participated in the study. The refractive status was determined using an open-field autorefractor and the measurements of pupil size and intraocular pressure were also carried out. A variety of visual functions were measured binocularly for the children who are currently using atropine to control myopia progression and for those who don’t use. The side effects and reading performance were assessed by questionnaires and the DEM test.

**Results**

45.7 % of the subjects were myopic, of which 32 % were on regular atropine treatment. The atropine group had a significantly larger pupil size (*p* < 0.001) but a similar mean IOP compared with the control group. Reduced BCVAs were found in children who were given atropine compared to the non-atropine group both at distance (0.40 ± 0.31 vs. 0.27 ± 0.34 logMAR, *p* < 0.05) and at near (0.11 ± 0.16 vs. 0.06 ± 0.08 logMAR, *p* < 0.01). Poorer binocular functions of convergence, accommodation and stereopsis were observed in the atropine group (*p* < 0.001). Higher frequencies of blur while reading, asthenopia and words/lines skipping (*p* < 0.001) were found in children with atropine usage and more horizontal errors (5.12 ± 5.20 vs. 2.55 ± 3.88, *p* < 0.01) were also observed in the DEM test.

**Conclusion**

A child’s visual function and reading performance can greatly affected by atropine usage. The combination of using other means to control myopia should be considered (e.g., progressive addition lens) and their visual function and ocular health long-term monitored.

## O7 Increased time outdoors prevents the onset of myopia: evidence from randomised clinical trials

### Ian G Morgan^1,2^ (ian.morgan@anu.edu.au)

#### ^1^Division of Biochemistry and Molecular Biology, Research School of Biology, Australian National University, Canberra, Australia; ^2^State Key Laboratory of Ophthalmology and Division of Preventive Ophthalmology, Zhongshan Ophthalmic Center, Sun Yat-Sen University, Guangzhou, China

Since the discovery, less than 10 years ago, that increased time outdoors prevented the onset of myopia from both cross-sectional and longitudinal data [1], there has been considerable interest in assessing whether this approach can be used to control the current epidemic of myopia. In Guangzhou, we have carried out a 3-year, school-based, randomised clinical trial, with an intervention of 40 minutes of additional time outdoors per school day. This produced a 23 % reduction in new cases of myopia in 6-9 year-old children [2]. In parallel, a 1-year trial carried out in Kaohsiung, Taiwan, produced around 50 % reduction in incident cases of myopia by locking primary school children out of their classrooms during school recesses, giving potentially an additional 80 minutes per day [3]. This suggests a dose-response relationship consistent with that seen in epidemiological data. Two other school-based trials from China reported consistent effects [4,5], and one trial has reported that simply increasing light intensity in the classrooms produced a significant protective effect [6]. This effect is difficult to reconcile with the evidence on time spent outdoors. These trials provide proof of principle for the effectiveness of this approach, and the challenge is now to develop more effective interventions. In general, these trials have not demonstrated reduced myopia progression, but the existence of marked seasonal effects on myopia progression [7,8] suggest that it can also be regulated by environmental factors; possibly the same factors that regulate onset.

**References**

1. French AN, Ashby RS, Morgan IG, Rose KA. Time outdoors and the prevention of myopia. Exp Eye Res. 2013;114:58-68.

2. He M, Xiang F, Zeng Y, Mai J, Chen Q, Zhang J, et al. Effect of time spent outdoors at school on the development of myopia among children in China: A randomized clinical trial. JAMA. 2015;314:1142-8.

3. Wu PC, Tsai CL, Wu HL, Yang YH, Kuo HK. Outdoor activity during class recess reduces myopia onset and progression in school children. Ophthalmology. 2013;120: 1080-5.

4. Jin JX, Hua WJ, Jiang X, Yang JW, Gao GP, Fang Y, et al. Effect of outdoor activity on myopia onset and progression in school-aged children in northeast China: the Sujiatun Eye Care Study. BMC Ophthalmol. 2015;15:73.

5. Yi JH, Li RR. Influence of near-work and outdoor activities on myopia progression in school children. Zhongguo Dang Dai Er Ke Za Zhi. 2011;13:32-5.

6. Hua WJ, Jin JX, Wu XY, Yang JW, Jiang X, Go GP, et al. Elevated light levels in schools have a protective effect on myopia. Ophthalmic Physiol Opt. 2015;35:252-62.

7. Deng L, Gwiazda J, Thorn F. Children's refractions and visual activities in the school year and summer. Optom Vis Sci. 2010;87:406-13.

8. Donovan L, Sankaridurg P, Ho A, Chen X, Lin Z, Thomas V, et al. Myopia progression in Chinese children is slower in summer than in winter. Optom Vis Sci. 2012;89:1196-202.

## O8 Environmental risk factors and gene-environment interactions for myopia in the ALSPAC cohort

### Jeremy A. Guggenheim^1^, Rupal L. Shah^1^, Cathy Williams^2^

#### ^1^School of Optometry & Vision Sciences, Cardiff University, Cardiff, UK; ^2^School of Social and Community Medicine, University of Bristol, Bristol, UK

##### **Correspondence:** Jeremy A. Guggenheim (GuggenheimJ1@cardiff.ac.uk)

**Purpose**

The Avon Longitudinal Study of Parents and Children (ALSPAC) is a birth cohort study that recruited 14,541 pregnant women from the county of Avon, UK, in 1991-1992 and has followed the health and well-being of the mothers and children until the present day. Comprehensive information about lifestyle has been collected from questionnaires completed by the mother and her child, and the child’s refractive error has been assessed at ages 7, 10, 11, 12 and 15 years of age (non-cycloplegic autorefraction). High density genotyping has been carried out for approximately 10,000 mothers and 10,000 children.

**Methods and results**

Past studies revealed that (1) time spent outdoors at age 8-9 years and physical activity (quantified using an accelerometer attached at the waist and worn for 1 week) at age 11 years were independently associated with a reduced risk of incident myopia, and (2) there was no evidence that the association between time outdoors and reduced risk of myopia was mediated by greater vitamin D from sunlight exposure. Additional studies now suggest that spending time outdoors at an early age (3 to 8 years) cumulatively reduces the risk of incident myopia, which implies that increased time outdoors at any age within this range will be beneficial in reducing the risk of myopia. Past studies by the CREAM consortium and the 23andMe company have identified ~40 commonly-occurring genetic variants associated with refractive error. Analyses of longitudinal refractive error trajectories in ALSPAC children provide a rare opportunity to test for gene-environment (G x E) interactions involving these variants. In such analyses, there was scant evidence for interactions involving time outdoors, but some evidence of interactions between SNPs and time spent reading were seen. An interaction between genetic variants at the APLP2 gene locus and time spent reading was also observed.

**Conclusions**

Time outdoors at an early age (below age 8 years) is associated with a reduced risk of incident myopia independent of the reduced risk associated with time outdoors at age 8-9 years. For the genetic variants associated with susceptibility to myopia identified to date, there is greater evidence for interactions with time spent reading than with time spent outdoors.

## O9 Retinal metabolic profiling identifies declines in FP receptor-linked signaling as contributors to form-deprived myopic development in guinea pigs

### Jinglei Yang^1,2^, Peter S. Reinach^1,2^, Sen Zhang^1,2^, Miaozhen Pan^1,2^, Wenfeng Sun^1,2^, Bo Liu^1,2^, Fen Li^1,2^, Xiaoqing Li^1,2^, Aihua Zhao^3^, Tianlu Chen^3^, Wei Jia^3^, Jia Qu^1,2^, Xiangtian Zhou^1,2^

#### ^1^School of Ophthalmology and Optometry and Eye Hospital, Wenzhou Medical University, Wenzhou, Zhejiang, 325027, China; ^2^State Key Laboratory Cultivation Base and Key Laboratory of Vision Science, Ministry of Health and Zhejiang Provincial Key Laboratory of Ophthalmology and Optometry, Wenzhou, Zhejiang, 325027, China; ^3^Shanghai Key Laboratory of Diabetes Mellitus and Center for Translational Medicine, Shanghai Jiao Tong University Affiliated Sixth People’s Hospital, Shanghai 200233, China

##### **Correspondence:** Xiangtian Zhou (zxt@mail.eye.ac.cn)

**Purpose**

Systems biology platforms including genomics, transcriptomics and proteomics have provided important information about the mechanisms underlying myopia development. Metabolomics, which quantifies the numerous metabolites that make up the metabolome of a given cell or tissue, has not yet been widely used to study ocular biology. This procedure provides an additional benefit in that it directly reads out of the physiological state of a cell or tissue at a given point in time and can also identify disease biomarkers. Here, we applied gas chromatography-time of flight mass spectrum (GC-TOF) analysis to explore the retinal metabolic profile of form-deprived myopic guinea pigs at different time points in the hope of providing key information about metabolic changes contributing to myopia development.

**Methods**

1. Retinal metabolite profiles were obtained using GC-TOF from guinea pigs including controls, 3 days and 2 weeks after form deprivation. Multi-dimensional PLS-DA models were used to discriminate retinal profiles in form-deprived and normal guinea pigs and identify important metabolites in pathways contributing to myopia development.
2. To determine whether alterations in metabolic pathways involving arachidonic acid (ARA, C20:4) are associated with refractive development, a prostaglandin F receptor(FP) agonist, Latanoprost, and an antagonist, AL8810, were injected into the sub-bulbar conjunctival space under local surface anesthesia. All injections were executed under dim red light in the right eye once daily (at 9:00 AM) for four weeks growing in normal visional environment or two weeks under form deprivation.

## Results

1. In PLS-DA models, retinal metabolic signatures in guinea pigs after 3 days and 2 weeks of form deprivation were different from those in normal controls. Furthermore, in a scores plot, the displacement between the form deprived samples and normal controls was associated with the degree of myopia development.
2. Octadecanoic acid (C18:0), octadecenoic acid (C18:1), arachidonic acid (ARA, C20:4) and DHA were at lower levels in retinas from guinea pigs 3 days and 2 weeks after form deprivation compared with controls, suggesting downregulation of ARA-associated metabolic pathways during myopia development.
3. Latanoprost-induced FP receptor activation had no effect on normal refraction development but inhibited FDM. Inhibition of FP receptors by AL8810 induced more myopia in both a normal visual and form deprivation environment.

**Conclusions**

During myopia development, ARA biosynthesis is downregulated based on declines in ARA concentration, its precursors and derivatives. These declines are consistent with the effects of a FP receptor agonist and antagonist. It is thus proposed that retinal PGF levels and FP receptor signaling pathway undergo downregulation during myopia development, which may be key to myopia mechanisms. These findings suggest that metabolomics is an effective tool to get insights into myopia onset.

## O10 The study of peripheral refraction in moderate and high myopes after one month of wearing orthokeratology lens

### Jun Jiang, Haoran Wu, Fan Lu

#### Eye Hospital of Wenzhou Medical University, Wenzhou, Zhejiang, 325027, China

##### **Correspondence:** Fan Lu (lufan@mail.eye.ac.cn)

**Purpose**

To evaluate the effects of using single-vision spectacles for residual refractive errors on retinal peripheral refraction of moderate and high myopic adolescents who were wearing orthokeratology lens at night.

**Methods**

Twenty moderate and high myopic adolescents with spherical equivalent over -5 diopters were recruited. Their refractive errors were not fully corrected after 1-month of wearing orthokeratology lens. The residual myopia was evaluated by cycloplegic subjective refraction and then corrected with single-vision spectacles. Visual acuity and peripheral refraction were measured respectively in the naked eyes, in the eye wearing orthokeratology lens and in the eye wearing single-vision spectacles.

**Results**

Relative peripheral refractions (RPREs) of the naked eye and the eye with spectacles showed significant myopic defocus at 10° and 20° in the nasal and temporal visual fields. RPREs of the eye wearing orthokeratology lens had slight myopic defocus or hyperopic shifts at 10° and 20° in the nasal and temporal visual field.

**Conclusions**

Although wearing orthokeratology lens in the daytime can correct residual refractive errors for high myopes, it may result in the decrease of myopia defocus or even a hyperopic shift in RPREs. Based on the hypothesis of peripheral refraction theory for myopia control, the spectacles may be a better choice than the daily-wear orthokeratology lens for correcting residual refractive errors in high myopes.

## O11 Axial length of school children around the Earth’s equatorial area and factors affecting the axial length

### Kazuo Tsubota^1^, Hiroko Ozawa^1^, Hidemasa Torii^1^, Shigemasa Takamizawa^2^, Toshihide Kurihara^1^, Kazuno Negishi^1^

#### ^1^Department of Ophthalmology, Keio University, School of Medicine, Shinjuku-ku, Tokyo, 160-8582, Japan; ^2^General Medicine, Ichikawa Medical Center, Urayasu City, Chiba, 279-0001, Japan

##### **Correspondence:** Kazuo Tsubota (tsubota@z3.keio.jp)

**Purpose**

The purpose of the present study was to examine the factors that influence axial length of Brazilian school children.

**Methods**

This retrospective study included 158 eyes of 158 Brazilian children (age range, 6-18 years; mean age, 10.2 ± 3.0 [standard deviation] years) at Professor Antonio Monteiro’s School located in Aracati City, Ceara, Brazil, who took a medical examination in August 2013. To identify the factors affecting axial length, we performed simple linear regression and multiple regression analyses (stepwise variable selection for regression), in which the covariates were age, sex, height, weight, dental health examination results, parental myopia, time playing outside or inside (reading, studying, watching television, playing video games), quality and regularity of sleep, and dietary habits.

**Results**

The average visual acuity (logMAR) was 0.12 ± 0.22, and axial length was 22.83 ± 0.75 mm. Multiple regression analysis in all cases showed that the axial length was significantly associated with gender, snacking between meals, height, and treatment for dental caries, and these results suggested being female, having a lower frequency of taking a snack, shorter stature, and good oral hygiene resulted in shorter axial length. Separating by age, the multiple regression analysis showed for age 6 & 7 years: female; for age 8 & 9 years: female with less time watching television, fewer dental caries, and less fruit consumption; for age 10 to 12 years: fewer dental caries; and for age 13 to 18 years: female with less snack consumption, have shorter axial lengths (P < 0.05).

**Conclusions**

The average axial length of Brazilian school children was shorter than previously reported in the same age groups in Asia. This study showed that axial length was correlated with gender, oral hygiene and dental care, duration of watching television or environmental factors such as frequency of eating snacks and fruits.

## O12 Processing of defocus in the chicken retina by retinal ganglion cells

### Klaus Graef^1,2^, Daniel Rathbun^1,2,3^, Frank Schaeffel^1^

#### ^1^Ophthalmic Research Institute, University of Tuebingen, 72076 Tuebingen, Germany; ^2^Bernstein Center for Computational Neuroscience, Tuebingen, 72076 Tuebingen, Germany; ^3^Werner Reichardt Centre for Integrative Neuroscience, Otfried-Mueller-Straße 25, Tuebingen, 72076 Tuebingen, Germany

##### **Correspondence:** Klaus Graef (Klaus.Graef@gmail.com)

**Purpose**

To find out whether chicken retinal ganglion cells are involved in the detection of defocus and its sign.

**Methods**

Two hundred and twenty-three retinal ganglion cells (RGC) were recorded with a 8x8 multi-electrode array (MEA, MultiChannel Systems, Reutlingen, Germany). The superfused retina was stimulated by projecting a color movie from RGC side via a Badal optical system. A movie was chosen to include motion cues. The Badal system ensured that image magnification remained constant for different amounts of defocus, provided in 2 D steps between -6 D and +6 D. Raw data recordings were made using MC_Rack (MultiChannel Systems). The resulting files were merged and analysed with Offline Sorter (Plexon Inc., Dallas, USA). A fundamental control to our data analysis was that also randomly generated data were studied. They never resulted in any significant differences.

**Results**

It was found that some ganglion cells responded to defocus and some even to its magnitude and sign. While some cells showed significant or highly significant differences in their activity conditioned on the sign of defocus, other cells showed a sign dependent response that followed the magnitude of the defocus.

Pooling cells that showed a significant difference in one defocus condition compared to the other conditions resulted in groups of cells which also showed significant differences in the named conditions. This is true even though the selection specifically ignored if the significance was a result of a higher or lower response rate.

Interestingly, selecting for positive defocus usually resulted in a loss of the significance in the group, even though each individual cell showed a significant difference in the response pattern of positive defocus versus the other conditions, hinting at a possible loss of the information of positive defocus on the level of the ganglion cells.

**Conclusions**

Our experiments suggest that the information on the sign and magnitude of defocus is represented in the RGC network. Up to about 15 % of the RGC population are sensitive to defocus, with a strong emphasis on negative defocus. Previous experiments (involving optic nerve section or TTX application to block RGC action potentials) had suggested that RGCs are not necessary for local retinal control of eye growth; amacrine cell output seemed sufficient. However, it could also be that the RGCs form local feedback circuits with the amacrine cells and that these networks are necessary to extract the sign of defocus from the retinal image.

## O13 Blue SAD light protects against form deprivation myopia in chickens, by local signaling within the retina

### Ladan Ghodsi^1^, William K. Stell^2^

#### ^1^O’Brien Centre for the Bachelor of Health Sciences, Cumming School of Medicine, University of Calgary, Calgary, Alberta, Canada; ^2^Departments of Cell Biology and Anatomy, and Surgery, Cumming School of Medicine, University of Calgary, Calgary, Alberta, Canada

##### **Correspondence:** William K. Stell (wstell@ucalgary.ca)

**Purpose**

Outdoor light may protect against myopia (near-sightedness), a developmental disorder of childhood and adolescence and a major cause of visual impairment. Sunlight is richer in blue than most indoor lights, and strong blue light activates the retinal melanopsin system. Therefore, we tested the hypothesis that moderately intense blue light inhibits the development of myopia, by activating melanopsin in intrinsically photoreceptive retinal ganglion cells (ipRGC).

**Methods**

Treated (T) eyes of chicks (P7-9) were goggled to induce form-deprivation myopia (FDM); control (C) eyes were open. Chicks were reared under light for treating Seasonal Affective Disorder (SAD light; Philips HF3332/60 GoLITE BLU, λ_max_ = 474 nm peak, half width = 25 nm) or ordinary fluorescent room light. We injected into T-eyes: tetrodotoxin (0.7 μg in 7 μL) to block signaling by ipRGCs; opsinamide AA92593 (100 μM) to block melanopsin-based signaling [1]; blockers of glutamatergic transmission (CNQX, L-AP4, MK-801) to block rod and cone signaling; and spiperone (0.5 mM) to block dopamine D2-R; in 20 μL vehicle. C-eyes received vehicle alone. We measured refraction and axial length of T- and C-eyes, and assessed ipRGC signaling by the pupillary light response (PLR).

**Results**

Full-intensity SAD light (~700 lux) for ≥1.5 hr reduced the FDM (excessive elongation, myopia) that developed in room light (≤500 lux). TTX did not block this effect, yet it blocked the PLR; but AA92593 did not, even in the presence of blockers of glutamatergic transmission. Spiperone abolished SAD light’s protective effect against FDM.

**Conclusions**

AA92593 appears not to affect melanopsin signaling by ipRGCs in chicks, although it does so in mice. Our data suggest that SAD light inhibits myopia via a local mechanism that involves dopamine acting via retinal D2-like receptors. SAD light could be a cost-effective therapy to inhibit myopia onset and progression, especially where exposure to sunlight is limited.

**References**

1. Jones KA, Hatori M, Mure LS, Bramley JR, Artymyshyn R, Hong S, et al. Small-molecule antagonists of melanopsin mediated phototransduction. Nat Chem Biol. 2013;9(10):630–5.

## O14 Contributions of ON and OFF pathways to emmetropization and form deprivation myopia in mice

### Machelle T. Pardue^1,2^, Ranjay Chakraborty^1,3^, Han na Park^1,3^, Curran S. Sidhu^3^, P. Michael Iuvone^3,4^

#### ^1^Atlanta VA Center for Visual and Neurocognitive Rehabilitation, Decatur, GA, 30033, USA; ^2^Department of Biomedical Engineering, Georgia Institute of Technology, Atlanta, GA, 30332, USA; ^3^Ophthalmology, Emory University, Atlanta, GA 30322, USA; ^4^Pharmacology, Emory University, Atlanta, GA 30322, USA

##### **Correspondence:** Machelle T. Pardue (mpardue@emory.edu)

**Purpose**

The exact visual signals that modulate visually-driven eye growth are not known. ON and OFF pathways are essential for processing information about contrast sensitivity and illumination. In addition, ON pathway stimulation increases retinal dopamine levels, a key modulator of myopic eye growth. To determine the role of ON and OFF retinal pathways in emmetropization and form-deprivation myopia, we examined mouse models with defects in these pathways.

**Methods**

Refractive development of mutant mice with ON (nob^nyx/nyx^ or mGluR6-/-) or OFF (Vsx1-/-) pathway defects and their wild-type (WT) controls were measured from 4 to 12 weeks of age. Mice were raised under normal visual environment or received a monocular diffuser goggle over the right eye anchored by a head-mounted pedestal. Weekly or biweekly measurements were taken using an automated photorefractor, keratometer, or optical coherence interferometer for ocular biometry. At the end of the study, retinas were analyzed for dopamine and DOPAC levels using HPLC.

**Results**

Mice with ON pathway mutations had disrupted refractive development across age with nob^nyx/nyx^ mice showing more hyperopic refractions and mGluR6-/- having more myopic refractions compared to their respective age-matched WT controls. In response to form deprivation, both ON pathway mutants had increased susceptibility to myopia (5-6 D myopic shift after 2 weeks). In contrast, the Vsx1-/- mice showed no significant differences in refractive development or response to form deprivation compared to WT mice. Dopamine metabolism was lower in the ON pathway mutants, but not the Vsx1-/- mice.

**Conclusions**

As predicted, ON pathway mutants have lower retinal dopamine levels. Both ON pathway mutant strains had increased susceptibility to form deprivation myopia, perhaps related to the low dopamine. These results suggest that ON pathway transmission may have a greater role in modulating visually-driven eye growth than OFF pathway stimulation.

## O15 Response of the human choroid to defocus

### Michael J Collins (m.collins@qut.edu.au)

#### School of Optometry and Vision Science, Queensland University of Technology, Brisbane, Queensland, 4059, Australia

There is substantial evidence that optical defocus is an important visual factor that guides the growth of the eye. Defocus can influence eye length by modulating changes in both scleral growth and choroidal thickness in various animal models. These changes lead to a movement of the retina toward the image plane. Changes in choroidal thickness in response to defocus can occur quickly and precede longer term changes in eye growth that are driven by scleral remodelling.

The use of high-resolution, precise methods (interferometry and optical coherence tomography) for measuring human eye dimensions has led to the finding that defocus can also lead to short-term changes in axial length (the axial distance from the anterior cornea to the retinal pigment epithelium) and choroidal thickness of human subjects. Similar to other animal species, human choroidal thickness and axial length shows bi-directional changes in response to both hyperopic and myopic defocus. These changes can occur quickly, with just a few minutes of exposure to defocus required to produce a response. When the defocus is imposed for a day, it significantly disrupts the normal diurnal rhythms in choroidal thickness and axial length with predictable patterns of change depending on the sign of the defocus.

This rapid response of the choroid provides a potentially important tool to understand the optical mechanisms that can influence eye growth.

## O16 What can RNA sequencing tell us about myopic sclera?

### Nethrajeith Srinvasalu^1^, Sally A McFadden^2^, Paul N Baird^1^

#### ^1^Centre for Eye Research Australia, University of Melbourne, Melbourne, Victoria, Australia; ^2^School of Psychology, University of Newcastle, Callaghan, Newcastle, NSW, Australia

##### **Correspondence:** Paul N Baird (pnb@unimelb.edu.au)

The outer coat of the eye, the sclera, is a fibrous connective tissue that provides mechanical strength to the eye. It maintains the ocular shape during normal growth and withstands ocular expansion in response to changes in intra-ocular pressure. However, the development of myopia is characterised by an increase in eye size, which indicates that the sclera must therefore undergo a series of changes to facilitate this excessive ocular growth. A better understanding of the biology and gene expression changes that exists within the sclera when myopic changes occur may thus provide novel avenues for intervention strategies. We know that the sclera is essentially a fibrous connective tissue, rich in extracellular matrix. Similar to other connective tissues, the scleral extracellular matrix is composed of collagens, proteoglycans, protease enzymes and is maintained by a resident population of scleral cells. The turnover of these matrix components and activity of scleral cells maintain the scleral integrity and thus the eye size. This presentation will discuss what we know about scleral structure, its biochemistry and how these changes are modulated during myopia development. We will also discuss future developments through the use of next generation sequencing technologies that will enable us to identify gene expression changes in the sclera at the genome level.

## O17 Overview of dopamine, retinal function, and myopia

### P. Michael Iuvone (miuvone@pharm.emory.edu)

#### Departments of Ophthalmology and Pharmacology, Emory University School of Medicine, Atlanta, GA, 30322, USA

Dopamine is an important retinal neuromodulator that regulates multiple aspects of light-adapted vision, retinal circadian rhythms, and refractive development. The presentation will describe and discuss the anatomy and physiology of retinal dopamine amacrine/interplexiform cells, and their regulation by light. The role of ON pathways and melanopsin in the regulation of retinal dopamine neurons will be discussed, as will the roles of dopamine in regulating visual function in healthy and diseased retinas. Early studies showed that reduced dopamine contributes to the development of form-deprivation myopia in chickens and monkeys. These studies will be briefly reviewed, followed by a review of recent studies with mouse models suggesting roles of dopamine in normal refractive development and sensitivity to form-deprivation myopia.

## O18 The eye as a "robust" optical system and myopia

### Pablo Artal (pablo@um.es)

#### Laboratorio de Óptica, Universidad de Murcia, Campus de Espinardo, 30100 Murcia, Spain

In this presentation, I will revise the optical properties of the human eye. In particular, I will concentrate on those aspects that may have an interest for the development of myopia. The human eye is a relatively simple optical instrument. This limits the quality of the retinal image affecting vision and may affect the development of refractive errors. However, it is also an extremely robust system. It can be considered as an aplanatic optical instrument, corrected of spherical aberration and coma. The relationship between the eye’s quality, the aplanatic properties and refractive error will be also discussed. Another important aspect with possible relevance for myopia is the quality of the eye at the periphery. Some results on this topic in eyes with different refractive errors and also a comparison of normal and pseudophakic eyes and will be also presented.

## O19 Effect of discontinuation of orthokeratology lens wear on axial elongation in children

### Pauline Cho, SW Cheung

#### School of Optometry, The Hong Kong Polytechnic University, Hong Kong SAR, China

##### **Correspondence:** pauline.cho@polyu.edu.hk

**Purpose**

To investigate the effect of discontinuation of orthokeratology (ortho-k) treatment on eyeball elongation in children who had completed a 2-year myopic control study.

**Methods**

Three groups of subjects were recruited: Spectacle-wearing and ortho-k subjects who continued with their respective treatments for the duration of the project, and ortho-k subjects who stopped lens wear for 6 months and then resumed ortho-k lens wear for another 6 months, with about a 1 month stabilization period between changes in treatment. Axial length was measured using the IOLMaster.

**Results**

A total of 53 subjects were enrolled. Axial length elongated faster (p = 0.003) in the first 6 months when lens wear was terminated (n = 18), after the 2-year myopic control study, compared to the other two groups of subjects (n = 16, 19). However, when controlled for age and initial axial length, no difference between groups was found (ANCOVA; p > 0.05).

**Conclusion**

Increase in axial length after ceasing ortho-k lens wear was not significant if controlled for age and initial axial length.

Results have been presented at EurOK (European Section of the International Academy of Orthokeratology) in Budapest in July 2015.

## O20 Myopia prevention in Taiwan

### Pei-Chang Wu (wpc@adm.cgmh.org.tw)

#### Department of Ophthalmology, Kaohsiung Chang Gung Memorial Hospital, and Chang Gung University College of Medicine, Kaohsiung, Taiwan

The Taiwan Student Vision Care Program (TSVCP) promoted by the Ministry of Education has been carried out for 3 decades in Taiwan. The myopia prevalence rapidly increased to a very high level and therefore myopia prevention is always an important factor in the program [1]. Due to the lack of evidence-based protective factors, myopia prevalence did not decrease even when a lot of effort had been taken before. After outdoor activities was discovered as an important myopia protective factor and was implemented in the TSVCP, there was a breakthrough in the last 3 years [2]. The nationwide visual acuity screen of elementary school showed the poor vision rate (uncorrected visual acuity 20/25 or less) declined unprecedentedly. This change would contribute to the "Prevent myopia with outdoor activities" strategy in the TSVCP.

**References**

1. Lin LL, Shih YF, Hsiao CK, Chen CJ. Prevalence of myopia in Taiwanese schoolchildren: 1983 to 2000. Ann Acad Med Singapore. 2004;33:27-33.

2. Wu PC, Tsai CL, Wu HL, Yang YH, Kuo HK. Outdoor activity during class recess reduces myopia onset and progression in school children.. Ophthalmology. 2013;120:1080-5.

## O21 Alternatives to ultraviolet light and riboflavin for in vivo crosslinking of scleral collagen

### Quan V. Hoang^1^, Sally A. McFadden^2^

#### ^1^Department of Ophthalmology, Columbia University College of Physicians and Surgeons, New York, NY, 10032, USA; ^2^School of Psychology & Hunter Medical Research Institute, The University of Newcastle, Callaghan, NSW, 2308, Australia

##### **Correspondence:** Quan V. Hoang (quh9003@gmail.com)

Myopic progression occurs in up to 50 % of myopic patients. Scleral weakness and abnormal scleral collagen are possible etiologic factors. Collagen crosslinking was introduced as a possible treatment technique for corneal ectasias in the late 1990s, where riboflavin activated by ultraviolet-A (UVA) light is the most common approach toward increasing corneal stability. This technique has also been applied to the posterior sclera, in an attempt to strengthen biomechanically weak sclera in cases of progressive myopia and reduce axial eye elongation. However, scleral collagen crosslinking with riboflavin and UVA has faced challenges, including difficulty accessing the posterior sclera with a UV light source. Additionally, UV light exposure to the eye has been implicated in a number of deleterious effects. There is a major potential benefit of developing an alternative method for cross-linking that does not involve UV light. In this presentation we evaluate the alternative methods of achieving scleral collagen crosslinking, including the use of chemicals activated by safe, visible light, the use of non-light-activated chemical collagen crosslinking and the potential of these methods to be translated into therapeutic use.

## O22 Absence of intrinsically photosensitive retinal ganglion cells (ipRGC) alters normal refractive development in mice

### Ranjay Chakraborty^1,2^, Duk C. Lee^2^, Erica G. Landis^1,2^, Michael A. Bergen^2^, Curran Sidhu^1^, Samer Hattar^3^, P. Michael Iuvone^1,4^, Richard A. Stone^5^, Machelle T. Pardue^1,2,6^

#### ^1^Ophthalmology, Emory University School of Medicine, Atlanta, GA, 30307, USA; ^2^Rehab R&D Center of Excellence, Atlanta VA Medical Center, Decatur, GA, 30033, USA; ^3^Biology and Neuroscience, Johns Hopkins University, Baltimore, MD, 21218, USA; ^4^Pharmacology, Emory University, Atlanta, GA, 30322, USA; ^5^Ophthalmology, University of Pennsylvania School of Medicine, Philadelphia, PA, 19104, USA; ^6^Wallace H. Coulter Department of Biomedical Engineering, Georgia Institute of Technology, Atlanta, GA, 30332, USA

##### **Correspondence:** Ranjay Chakraborty (ranjay.chakraborty@emory.edu)

**Purpose**

ipRGCs regulate a range of non-image forming functions of light such as modulation of circadian activity and pupillary light reflex. Their intrinsic response to light influences intra-retinal signaling and retinal dopaminergic cell activity. This study examined the role of ipRGCs in refractive development of the eye under normal and form-deprived (FD) visual conditions, using a mutant mouse in which the ipRGCs are ablated by diphtheria toxin (*Opn4* ^*DTA/DTA*^).

**Methods**

Normal refractive development of *Opn4* ^*DTA/DTA*^ and age-matched C57BL/6 J wild-type (WT) mice, were measured every 2 weeks from 4 to 16 weeks of age. A separate cohort of mice were fitted with a monocular diffuser goggle over the right eye to induce FD starting at 4 weeks of age, and were tested weekly. Refractive error was measured using an automated photorefractor, corneal curvature with keratometry, and ocular parameters with spectral-domain optical coherence tomography.

**Results**

Under normal visual conditions, *Opn4* ^*DTA/DTA*^ mice (n = 12) had abnormal refractive development with significant myopic refractions than their WT counterparts (n = 10) at 4 weeks (mean refractive error ± SEM, +0.56 ± 0.48 D vs. +2.96 ± 0.35 D, p < 0.05), shifting towards more hyperopic refractions than WT controls by 16 weeks (*Opn4* ^*DTA/DTA*^: +7.82 ± 0.35 D, WT: +6.63 ± 0.37 D, p < 0.05). *Opn4* ^*DTA/DTA*^ mice exhibited significantly shorter axial length than WT animals (mean at 12 weeks, *Opn4* ^*DTA/DTA*^: 3.25 ± 0.01 mm; WT: 3.37 ± 0.01 mm, p < 0.001). After 4 weeks of FD, *Opn4* ^*DTA/DTA*^ mice (n = 8) showed a significant myopic shift (difference between goggled right and contralateral left eyes) of -2.74 ± 0.50 D (p = 0.001) compared to non-goggled control mice (n = 8, -0.16 ± 0.46 D). Goggled WT animals (n = 9) exhibited a similar magnitude of myopic shift to *Opn4* ^*DTA/DTA*^ animals after 4 weeks of FD (-2.96 ± 0.48 D, p < 0.05). Corneal curvature showed no significant differences between the two strains under either normal or FD conditions.

**Conclusions**

These findings suggest that presence of ipRGCs is important for refractive development under normal visual conditions, but not FD conditions in mice. Refractive development in *Opn4* ^*DTA/DTA*^ is similar in refractive profile and response to FD as mice lacking melanopsin (*Opn4* ^*-/-*^). Together, these findings suggest a central role for circadian rhythms, perhaps intrinsic to the retina, in refractive development.

## O23 Scleral micro-RNAs in myopia development and their potential as therapeutic targets

### Ravi Metlapally (metlapally@berkeley.edu)

#### School of Optometry, University of California, Berkeley, CA, 94720, USA

The sclera determines the final size of the eye, and guides the exaggerated ocular growth in myopia by undergoing molecular changes leading to extracellular matrix loss, thinning and biomechanical weakening. By inhibiting scleral molecular changes in myopia via devising therapies to promote natural matrix deposition and improve scleral strength, there is potential to resist ocular elongation and slow myopia progression. Micro (mi)- RNAs are small non-coding RNAs that play pivotal roles in molecular cell signaling networks and gene (messenger (m)- RNA) regulation, and thus in normal physiological as well as disease processes. They are potential therapeutic targets for myopia control since scleral miRNAs have been previously implicated in the regulation of normal ocular growth. The presentation will showcase evidence for scleral miRNA regulation in myopia development. Proof that scleral miRNAs can be manipulated to modulate gene expression and in turn affect cell signaling networks will also be presented. Findings will be discussed in the context of therapeutic approach for myopia control, targeting the sclera.

## O24 Effects of the long-wavelength filtered continuous spectrum on emmetropization in juvenile guinea pigs

### Ruiqin Li^1,2^, Qinglin Xu^2,3^, Hong Zhong^2,3^, Chenglin Pan^2,3^, Weizhong Lan^2^, Xiaoning Li^2,3^, Ling Chen^1,2^, Zhikuan Yang^1,2,3^

#### ^1^Aier School of Ophthalmology, Central South University, Changsha, Hunan, China; ^2^Optometry Visual Science Institute, Aier Eye Hospital Group, Changsha, Hunan, China; ^3^Changsha Aier Eye Hospital, Changsha, Hunan, China

##### **Correspondence:** Zhikuan Yang (13380071988@189.cn)

**Purpose**

To investigate the effects of long-wavelength filtered continuous spectrum on emmetropization in juvenile guinea pigs.

**Methods**

One hundred and three 2-week-old pigmented guinea pigs were randomly assigned to two groups of high (Hi, 4000 lux) and low (Lo, 400 lux) intensities. Each group was subdivided into broad spectrum Solux halogen light (HiBS, n = 12; LoBS, n = 12), 600 nm and above wavelength filtered continuous spectrum (Hi600F, n = 13; Lo600F, n = 14), 530 nm and above wavelength filtered continuous spectrum (Hi530F, n = 12; Lo530F, n = 13), 480 nm and above wavelength filtered continuous spectrum (Hi480F, n = 14; Lo480F, n = 13) under a 12:12 h light/dark cycle for 6 weeks. Refractive error, corneal curvature radius, and axial dimensions were determined by cycloplegic retinoscopy, photokeratometry, and A-scan ultrasonography every 2 weeks, respectively. Changes in the ocular parameters and refractive errors relative to the baseline of the 206 eyes were calculated and compared among different groups by one-way ANOVA and Fisher’s least significant difference (LSD) post hoc test.

**Results**

After 6 weeks of exposure, guinea pigs under high intensity showed similar changes in the corneal curvature radius, anterior chamber depth, lens thickness, vitreous chamber depth (HiBS: 0.32 ± 0.05 mm; Hi600F: 0.30 ± 0.04 mm; Hi530F: 0.30 ± 0.04 mm; Hi480F: 0.31 ± 0.06 mm, P > 0.05) and refractive error (HiBS: 0.33 ± 1.55 D; Hi600F: 0.43 ± 1.51 D; Hi530F: 0.43 ± 1.54 D; Hi480F: 0.34 ± 1.38 D, P > 0.05). This was also true for the changes in the groups under low intensity except the increase of the vitreous chamber depth, although the broad spectrum group showed myopic shift while the other 3 filtered groups experienced hyperopic shifts (LoBS: -0.18 ± 1.38 D; Lo600F: 0.41 ± 1.35 D; Lo530F: 0.51 ± 1.49 D; Lo480F: 0.74 ± 1.14 D, P > 0.05).The broad spectrum group had a faster increase in vitreous chamber depth than the other 3 filtered ones (LoBS: 0.38 ± 0.06 mm VS Lo600F: 0.34 ± 0.06 mm; Lo530F: 0.34 ± 0.06 mm; Lo480F: 0.32 ± 0.05 mm, P < 0.05), but there were no significant differences among the 3 filtered groups.

**Conclusions**

Under a certain intensity, the long-wavelength filtered continuous spectrum seems to play a positive role in modulating the ocular growth in juvenile guinea pigs.

## O25 Ocular and environmental factors associated with eye growth in childhood

### Scott A. Read (sa.read@qut.edu.au)

#### School of Optometry and Vision Science, Queensland University of Technology, Brisbane, Queensland, 4059, Australia

Recent advances in measurement technology have improved our ability to quantify a range of ocular components and environmental exposures relevant to myopia. In particular, environmental sensor technology now allows the dense sampling of personal ambient light exposure data, and advances in ocular imaging, such as developments in optical coherence tomography (OCT) enables high resolution measures of the choroid to be captured in human subjects. The detailed, objective information produced by these non-invasive measurement technologies has the potential to provide important new insights into the complex array of factors underlying eye growth and myopia development and progression in childhood.

Wearable light sensors and enhanced depth imaging OCT were both employed in a prospective, observational longitudinal study examining factors associated with eye growth in myopic and non-myopic children. Personal light exposure, choroidal thickness and axial eye growth were quantified in 101 children over an 18-month period. A significant association was found between objectively measured personal daily ambient light exposure and eye growth (independent of refractive status), consistent with greater light exposure protecting against rapid growth of the eye in childhood. Variations in the thickness of the choroid also appeared to be closely linked to the growth of the eye, with choroidal thinning typically being associated with more rapid eye growth, and choroidal thickening with a slowing of eye growth in childhood. The implications of these findings for our understanding of human eye growth regulation, along with their potential importance for our understanding of myopia control interventions will be discussed.

## O26 Overview- prevention and prediction of myopia and pathologic myopia

### Seang-Mei Saw^1,2^ (seang_mei_saw@nuhs.edu.sg)

#### ^1^Myopia Research Unit, Singapore Eye Research Institute, Singapore, Singapore; ^2^Saw Swee Hock School of Public Health, National University of Singapore, Singapore, Singapore

There is an epidemic of high myopia in Asia and pathologic myopia will become a huge public health problem in urban aging cities such as Singapore, leading to decreased vision or quality of life. The prevalence of pathologic myopia from population-based studies varies from 0.9 to 3.1 %. The META-PM international consortium has classified myopia into 5 categories. The prevalence of PM is likely to increase drastically in older adults over the next few decades in Singapore, largely contributed by the ageing/generational effect. Currently, 83 % of young male adults and about 30 % in the adult and older adult populations (40 to 80 year olds) in Singapore are myopic. In 30 years, we predict that 83 % of older 40 to 50 year olds will be myopic. Children with early onset myopia may be at risk of high myopia and visually disabling pathologic myopia lesions later in life. The modification of at risk behavior such as lack of outdoor time may prevent or delay the onset of early myopia and reduce the risk of high myopia in later adulthood. Our proposed new FitSight watch to increase outdoor time in children is an entirely novel non-invasive, user-friendly treatment system for myopia prevention.

## O27 New insights into the roles of retinal dopamine in form-deprivation myopia and refractive development in C57BL/6 mice

### Shi-Jun Weng^1^, Xiao-Hua Wu^1^, Kang-Wei Qian^1^, Yun-Yun Li^1^, Guo-Zhong Xu^1^, Furong Huang^2^, Xiangtian Zhou^2^, Jia Qu^2^, Xiong-Li Yang^1^, Yong-Mei Zhong^1^

#### ^1^Institute of Neurobiology, Institutes of Brain Science, State Key Laboratory of Medical Neurobiology and Collaborative Innovation Center for Brain Science, Fudan University, Shanghai, 200032, China; ^2^School of Ophthalmology and Optometry and Eye Hospital, Wenzhou Medical University, Wenzhou, Zhejiang, 325035, China

##### **Correspondence:** Shi-Jun Weng (sjweng@fudan.edu.cn)

Retinal dopamine is thought to play a key role in the signaling pathway regulating visually guided eye growth. The mouse has recently been used as a mammalian model for exploring the mechanisms underlying refractive development. In recent years, we have studied how retinal dopamine levels could be associated with form-deprivation myopia (FDM) and refractive development in mice. In a work published in IOVS early this year, we reported that in C57BL/6 mice, unlike previous results obtained in any other myopic animal models showing decreased retinal dopamine levels during FDM development, retinal concentrations of dopamine/DOPAC and vitreal DOPAC levels (an index of extracellular dopamine), were unaltered during the development of FDM, either in the daytime or at night, as assessed by HPLC. Moreover, neither dopaminergic cell density, dopaminergic process area size, nor retinal tyrosine hydroxylase (TH), dopamine transporter (DAT) levels, were significantly attenuated in deprived eyes, suggesting an intact retinal dopaminergic system in myopic eyes. Retinal dopamine levels are thus unlikely associated with FDM development in this mouse strain. Most recently, we further investigated effects of retinal dopaminergic system lesion induced by 6-hydroxydopamine (6-OHDA) on refractive development and FDM formation in this mouse strain. Intravitreal injections of 6.25 μg and 12.5 μg of 6-OHDA, which markedly reduced retinal dopamine levels for at least 31 days, but without producing a non-specific toxic effect on ERG a- and b-wave amplitudes, were chosen for all the experiments. With normal visual experience, the 6-OHDA-injected eyes became myopic relative to the fellow eyes, and the degree of myopic shifts was dose-dependent (around -2.8 D for 6.25 μg 6-OHDA and - 4.6 D for 12.5 μg 6-OHDA), suggesting that retinal dopamine levels are associated with normal refractive development in C57BL/6 mice. Furthermore, form-deprivation induced further myopic shifts in eyes with 6.25 μg 6-OHDA treatment, resulting in an overall shift comparable to that induced by pure form-deprivation treatment. However, in eyes treated with 12.5 μg 6-OHDA injection, form-deprivation failed to further change the myopia status. In conclusion, these results strongly suggest that the refractive development of C57BL/6 mice could be modified by two distinct mechanisms regarding dopamine dependence. While the development of FDM is mediated by a dopamine-independent signaling cascade, dopamine levels do affect normal refractive development. The effects generated by the two mechanisms, to a certain extent, are superimposable. Whilst this work provides new insights into the roles dopamine plays in myopia and refractive development, several remarks should be made: (1) Species differences might contribute to the sharp discrepancy concerning changes in retinal dopamine levels in myopic eyes between this and previous studies. Any conclusions drawn from the present work only apply to the C57BL/6 mouse and cannot be expanded to other species without further experimental evidence. (2) It has yet to be determined whether unaltered retinal dopamine levels also hold true for mouse myopia models induced by treatments other than form-deprivation, such as in lens induced myopia (LIM) models of the mouse. (3) The unchanged retinal dopamine levels and the intactness of the dopamine system also do not necessarily imply that the dopamine system plays no role in myopia development of this mouse strain. Whether changes in dopamine receptor activity, instead of dopamine levels, may be associated with myopic development remains to be explored.

## O28 The effects of the adenosine antagonist, 7-methylxanthine, on refractive development in rhesus monkeys

### Earl L Smith III^1,2^, Baskar Arumugam^1,2^, Li-Fang Hung^1,2^, Lisa A. Ostrin^1,2^, Klaus Trier^3^, Monica Jong^2^, Brien A. Holden^2^

#### ^1^University of Houston College of Optometry, Houston, TX, 77204, USA; ^2^Brien Holden Vision Institute, Sydney, Australia; ^3^Trier Research Laboratories, Tingskiftevej 6, 2900 Hellerup, Denmark

##### **Correspondence:** Earl L Smith III (esmith@uh.edu)

**Purpose**

Recent studies in rabbits and ongoing clinical trials in children suggest that systemic administration of the adenosine antagonist, 7-methylxanthine (7-MX) retards myopia progression. The aim of this study was to determine whether 7-MX influences lens-induced myopia in rhesus monkeys.

**Methods**

Starting at 3 weeks of age, infant monkeys (n = 16) were reared with -3D lenses over their treated eyes and plano lenses in front of their control eyes; three of the lens-reared monkeys were also given 100 mg/kg of 7-MX by mouth two times a day. Control data were obtained from 34 normal monkeys. Refractive status, corneal power and axial dimensions were assessed every 2 weeks throughout the lens rearing period. Choroid thickness was measured in a subset of the lens-reared monkeys using OCT.

**Results**

In the animals reared on a typical laboratory diet, the imposed hyperopic defocus consistently produced compensating myopic changes in the treated eyes. In contrast, none of the 7-MX treated monkeys developed compensating anisometropia. At the end of the lens-rearing period, the average anisometropia (treated eye vs. control eye) was significantly smaller in the 7-MX treated monkeys (+0.15 ± 0.20 D vs. -2.22 ± 1.09 D; p = 0.003). In addition, the control eyes of the 7-MX monkeys were axially more hyperopic than the eyes of the normal monkeys (Rx: +5.56 D vs. +2.50 D, p = 0.008; VC: 9.27 ± 0.19 mm vs. 9.82 ± 0.30 mm, p = 0.004). The choroids in both eyes of the 7-MX monkeys were also thicker than those in the eyes of the lens-reared monkeys that were not treated with 7MX (7-MX -3 D, 183.3 ± 5.73 μm vs. -3 D, 119.7 ± 23.2 μm, p = 0.02; 7-MX control eye, 176.8 ± 6.5 μm vs. -3 D control eye, 129.9 ± 14.7 μm, p = 0.01).

**Conclusions**

The results demonstrate that the daily systemic administration of 7-MX increases choroidal thickness and reduces overall axial growth rates while blocking the compensating myopia normally produced by imposed hyperopic defocus in primates.

## O29 Application of SWATH™ based next generation proteomics (NGP) in studying eye growth: opportunities and challenges

### Thomas Chuen Lam^1^, Samantha Shan^1^, Bing Zuo^1,2^, Sally A. McFadden^3^, Dennis Yan-yin Tse^1,3^, Jingfang Bian^1^, King-Kit Li^1^, Quan Liu^2^ and Chi-ho To^2^

#### ^1^Laboratory of Experimental Optometry, Centre for Myopia Research, School of Optometry, The Hong Kong Polytechnic University, Hung Hom, Hong Kong, China; ^2^State Key Laboratory of Ophthalmology, Zhongshan Ophthalmic Center, Sun Yat-Sen University, Guangzhou, China; ^3^School of Psychology, The University of Newcastle, Faculty of Science and IT, Callahgan, New South Wales, Australia

##### **Correspondence:** Thomas Chuen Lam (Thomas.c.lam@polyu.edu.hk)

Myopia is a multifactorial ocular condition that is characterized by an excessively elongated eyeball. Previous animal studies have shown that myopic eye growth is regulated by complex biochemical signalling cascades that take place locally in the retina, choroid and sclera. Conventional approaches of studying these biochemical cascades usually target single or a few molecular candidates at a time. They reveal limited global information on the interplay between multiple pathways that orchestrate myopic eye growth. The proteomic approach is a high throughput technique that allows thousands of protein changes to be identified in one setting. Early attempts using the classical 2D gel electrophoresis technique and discovery proteomics have identified some novel proteins, which may be involved in myopic eye growth. However, the complexity of ocular proteomes has hindered a more comprehensive understanding of the mechanism of myopia development. Emerging advances in Mass Spectrometry (MS) with Data Independent Acquisition (DIA) allow identification and quantitative comparison of over thousands of retinal proteins across different biological samples in a highly sensitive and repeatable manner.

Applying SWATH™ MS in studying the retinal tissues from growing guinea pigs (Cavia porcellus) at 3 days old and at 21 days old, a comprehensive SWATH ion-library retina consisted of 3138 non-redundant proteins (at 1 % FDR) were established. 80 % of the proteins could be co-identified among all sample replicates to provide a large reservoir of protein candidates for quantification. Very similar protein translation modifications (PTMs) could also be detected from identified peptides across all biological groups. In contrast, only 63 % of proteins were commonly detected using the standardised shotgun approach. Through integrated group analysis in the OneOmics^TM^ platform, 48 unique proteins were found to be significantly expressed between the two time points. Pathway analysis indicated their involvement in a number of biochemical processes including development, metabolism and signal transduction. The advent of SWATH™ MS with integration of cloud-based bioinformatics provides a high-throughput platform that could contribute further to our basic understanding of myopia.

**Acknowledgements**

GRF 251006/14 M, GRF BQ-29 N (#562611), PolyU PhD Studentship (RTX2), University of Newcastle Grant G0900214.

## O30 How could emmetropization make use of longitudinal chromatic aberration?

### Timothy J. Gawne, John T. Siegwart, Jr., Alexander H. Ward, Thomas T. Norton

#### Department of Optometry and Vision Science, University of Alabama at Birmingham (UAB) Birmingham, AL, 35294, USA

##### **Correspondence:** Timothy J. Gawne (tgawne@gmail.com)

**Purpose**

Postnatally, mammalian eyes normally use refractive error to modulate their growth to achieve good focus, a process called emmetropization. However, the specific visual cues that the eye uses to regulate its growth remain unclear. The eye could detect Longitudinal Chromatic Aberration (LCA) and use the relative image contrast in long (red) and short (blue) wavelengths to guide eye growth. However, short-wavelength sensitive (SWS) photoreceptors (cones) are sparsely present in the retina, so it would not seem possible for the SWS cone system to judge the blue image sharpness (spatial contrast) with any precision. We hypothesized that when the blue wavelengths are in focus, the transient responses of SWS cones as an animal moves around, or moves its eyes, could be used as a proxy for blue image sharpness. Thus, flickering blue light might signal that the eye is too short and needs to grow. Conversely, red light with no blue should slow eye growth independent of flicker.

**Methods**

Juvenile tree shrews were placed in ambient light that varied in both chromatic content (red, 628 ± 10 nm, 325-700 human lux, or blue, 464 ± 10 nm, 241-500 human lux) and also temporal profile (steady, or flickering with sharp transients to mimic what happens on the back of the retina as an animal moves around: broadband from DC to over 100 Hz). The red light only excited LWS cones; the blue light excited SWS cones 1.56 times more strongly than the LWS cones. Refractive state was measured, often daily, with a Nidek autorefractor while the animals were awake. Axial component dimensions were measured with a LenStar optical biometer in awake animals at key points during the experimental period.

**Results**

Tree shrews developing in fluorescent colony lighting are initially hyperopic; the eye then grows rapidly so that at 24 Days of Visual Experience (DVE) they are only a diopter or so hyperopic. In contrast, all five tree shrews exposed to steady red background lighting remained or became strongly hyperopic. At the end of steady red light (14 hours red/10 hours dark) treatment at 24 DVE the group refraction was (7.0 ± 0.7 D), approximately 5.8 D above normals at that age. The depth of their vitreous chamber (2.65 ± 0.02 mm) was significantly shorter than the normals (2.79 ± 0.038 mm), indicating that refractive hyperopia occurred because the eye was short. When exposed to regular colony fluorescent lighting the hyperopia in all animals rapidly decreased toward age appropriate emmetropia, demonstrating that the red light exposure had not damaged the emmetropization mechanism. Similar results were obtained for animals exposed to steady red light: 4.7 ± 0.8 D, a lower hyperopia than in the steady red group but approximately 3.5 D above normals. Exposure to steady blue light did not, on average, have a strong effect on emmetropization. However, flickering blue light was myopiagenic: by the end of treatment the group average (-3.4 ± 1.7 D) was approximately 4.6 D myopic compared with the animals raised in colony lighting. The vitreous chamber depth of this group (2.94 ± 0.05 mm) was also significantly larger than normal. Preliminary data indicate that red light can significantly retard eye growth even if used for only part of the day, perhaps similarly to how brief periods of clear vision can protect against the myopiagenic effect of minus lenses. Red light also appears to be effective in slowing eye growth in older animals (35 DVE), which is closer to the age at which human children develop myopia. However, red light does not completely stop the myopiagenic effect of wearing minus lenses. Finally, preliminary studies with both red and blue light present suggest that the relative flicker between red and blue could also be important.

**Conclusions**

Regardless of whether the developing eye indeed uses LCA to help guide its growth, we have found stimuli that have substantial effects on refractive development. The magnitude of these effects suggest that we are engaging the fundamental mechanisms of emmetropization. Monochromatic red light might potentially be developed into a method of slowing the progression of myopia in human children.

## O31 Balance effect of dopamine D1 and D2 receptor subtype activation on refraction development

### Xiangtian Zhou (zxt@mail.eye.ac.cn)

#### School of Ophthalmology and Optometry, Wenzhou Medical University, Wenzhou, Zhejiang, 325027, China

We and others found that dopamine (DA), an important endogenous neuromodulator in the retina, declines during experimental myopia development in animal models indicating that DA levels affect myopia development. Our group has carried out extensive and in depth studies in order to help clarify the role of DA in myopia development. Similar to previous reports in chicks, monkeys and some other species, the non-selective DA agonist apomorphine attenuated monocular development of form deprived myopia (FDM) in C57BL/6 mice and pigmented guinea pigs. Furthermore, either selective DA D1 receptor agonist or D2 receptor antagonist both inhibited spontaneous myopia in albino guinea pigs and FDM development in mice as well as pigmented guinea pigs. On the other hand, a DA D1 receptor antagonist or a D2 receptor agonist instead enhanced spontaneous myopia in albino guinea pigs and FDM development in pigmented guinea pigs. These results indicate that D1 receptor activation inhibits whereas D2 receptor activation promotes myopia development. In other words, the DA D1 receptor and D2 receptor activation exerts opposite effects on myopia development.

Compared to wild type (WT) littermates, DA D2 receptor knock out (KO) mice developed more hyperopia and FDM was partially inhibited, suggesting that D2 receptor inactivation attenuates myopia development. In WT mice, different doses of a D2 receptor agonist, quinpirole, had biphasic effects on FDM development: a high dose inhibited myopia development while a low dose instead promoted it. Importantly, this biphasic effect of quinpirole on FDM in WT mice disappeared in D2R KO mice, indicating that quinpirole acts on myopia development by a D2R-dependent mechanism. It is also possible that D2R autoreceptors may participate in these opposing effects. Meanwhile, a non-selective dopamine agonist, apomorphine, could still inhibit FDM in D2R KO mice, suggesting that D1 receptor activation also attenuates myopia development. In other words, both D2 receptor inactivation as well as D1 receptor activation could inhibit myopia development. Therefore, these two DA receptor subtypes together contribute to myopia development.

In summary, we found that D1 receptor activation promotes hyperopia development while D2 receptor activation promotes myopia development in different animals. During growth, these two receptor subtypes induce reciprocal cell signaling events controlling postnatal refraction and myopia development. Their regulation of visual growth and refraction is manifested in a balanced manner.

## O32 BMP gene expression changes in chick rpe in response to visual manipulations

### Yan Zhang, Yue Liu, Carol Ho, Eileen Phan, Abraham Hang, Emily Eng, Christine Wildsoet

#### School of Optometry, University of California at Berkeley, Berkeley, CA 94720, USA

##### **Correspondence:** Christine Wildsoet (Wildseot@berkeley.edu)

**Purpose**

A series studies were conducted to explore changes in BMP2, 4, and 7 gene expressions in the chick retinal pigment epithelium (RPE) during the altered ocular growth responses elicited by defocusing lenses and form-depriving diffusers.

**Methods**

Young White-Leghorn chickens were used in this study. Gene expressions of BMP2, 4, and 7 in RPE were examined in two experiments: 1) monocular +10 and -10 D lens treatment for 2 and 48 hours; 2) short treatment durations for 5, 15, 30, 60 minutes using a variety of visual manipulations including monocular -3, -10, +10, +20, and +30 D lenses, as well as diffusers. In a third experiment, gene expression in three different zones of RPE (central, middle, and peripheral zones: 3, 3-6, and 6-9 mm in radius, respectively) was examined with +10 and -10 D lens treatments for 2 and 48 hours. Protein expression of BMPs was also characterized in posterior ocular tissues using Western blots and immunohistochemistry.

**Results**

Gene expressions of BMP2, 4, and 7 showed optical defocus sign-dependent differences that also exhibited characteristic temporal and spatial features: 1) BMP2, 4 and 7 gene expressions were up-regulated in the RPE from eyes wearing +10 D lenses and down-regulated in those wearing -10 D lenses for 2 and 48 hours (treatment vs. fellow); 2) for very short exposures, defocus sign-dependent differences in gene expression patterns in RPE were lost; 3) defocus sign-dependent differences in gene expressions were preserved in the central and middle zones for BMP2 and BMP4, which both showed up-regulation with + 10 D lenses and down-regulation with -10 D lenses; for BMP7, only the central region showed up-regulation with +10 D lenses and no significant change in expression was observed with -10 D lenses in any of these three zones alone. In untreated chicks, BMP2, 4 and 7 proteins were found to be widely expressed in all posterior ocular tissues, including the retina, RPE, choroid, and sclera.

**Conclusions**

mRNA expression of BMP in chick RPE shows bidirectional, defocus, sign-dependent changes with distinctive spatial and temporal features, strongly suggesting regulatory roles for RPE-derived BMPs in early eye development.

